# A case report of Werner’s syndrome with bilateral juvenile cataracts

**DOI:** 10.1186/s12886-018-0873-4

**Published:** 2018-08-14

**Authors:** Chun-li Chen, Jia-song Yang, Xiang Zhang, Tian Tian, Rui Zeng, Guan-hong Zhang, Xin-guo Jia

**Affiliations:** 1 Department of Ophthalmology, Shengli Oilfield Central Hospital, Shandong Province, Dongying, 257000 China; 2 Department of Ophthalmology, Xin Hua Hospital Affiliate of Shanghai Jiao Tong University School of Medicine, Shanghai, 200092 China; 3 Vitreous & Retinal Department, Apex Eye Hospital, Xincai, 463500 Henan Province China; 4 Department of Ophthalmology, Tianjin Medical University Eye Hospital, Tianjin, 30000 China

**Keywords:** Werner’s syndrome, *WRN* mutation, Premature aging

## Abstract

**Background:**

To report a case of Werner’s syndrome with bilateral juvenile cataracts.

**Case presentation:**

Review of the clinical, laboratory, photographic, genetic testing of the patient.

A 26-year-old Chinese man presented with impaired vision in both eyes for more than a year. Anterior segment examination of both eyes revealed cataract. According to the ocular symptoms and systemic signs, including low body weight, a short stature, a bird-like face, atrophic and scleroderma-like skin, in addition to the juvenile cataracts, the clinical diagnosis of Werner’s syndrome was made. Next-generation sequencing identified a homozygous *WRN* mutation in this patient.

**Conclusions:**

The ocular and systemic findings in this patient in combination with the homozygous WRN mutation indicated the definitive Werner’s syndrome diagnosis.

## Background

Werner’s syndrome was first described by Werner in 1904; it is also known as adult premature aging syndrome, or progeria of adult. Werner’s syndrome is an autosomal recessive and rarely inherited disease characterized by onset of a prematurely aged-appearance (grey hair, scleroderma-like skin) typically starts in the 20–30s followed by age-related disorders like cataracts, diabetes mellitus, atherosclerosis, cancers, and osteoporosis [1, 2]. We present a case diagnosed with Werner’s syndrome confirmed by genetic testing, to improve doctors’ knowledge of this rare genetic disease.

## Case presentation

**Fig. 1:**
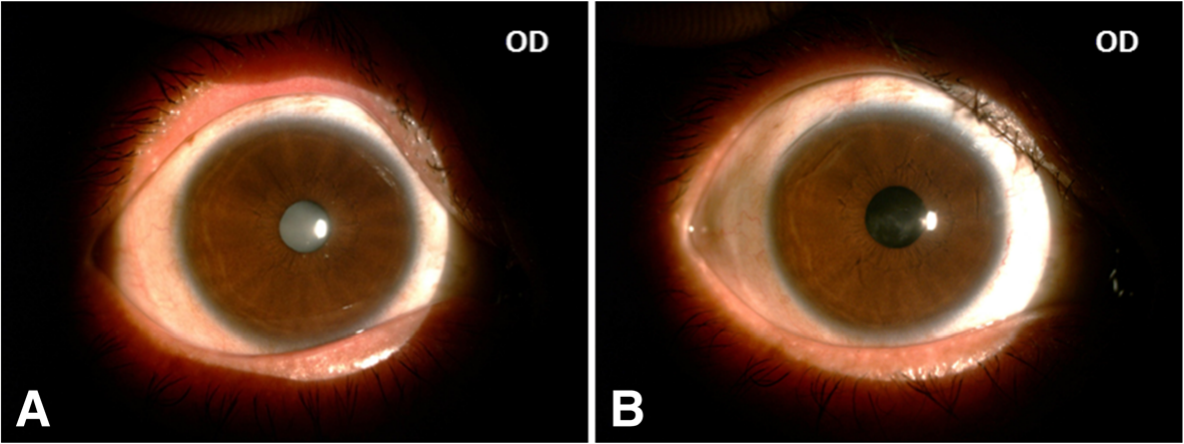
Photograph of the anterior segment of the right eye (OD). **a**, lens was opaque and cloudy; **b**, post-operative appearance showed the clear IOL in place. OD, right eye

**Fig. 2:**
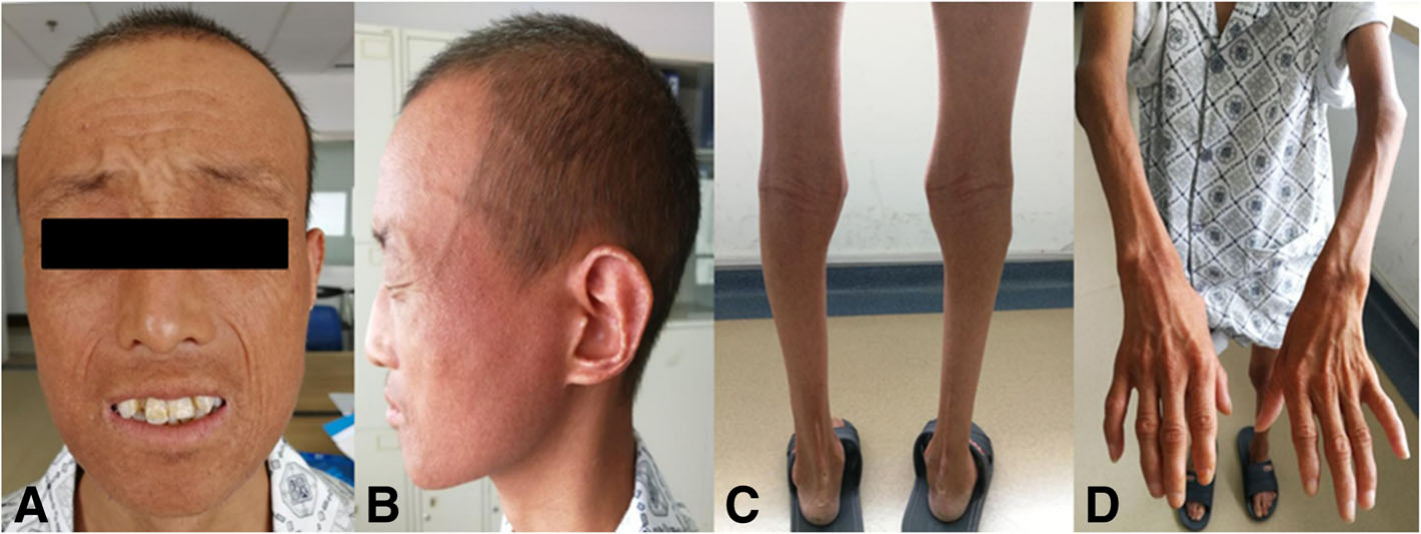
The appearance of the patient and his form extremely slight. **a**, beak-like nose and teeth abnormalities; **b**, Gray hair, bird-like face appearance; **c**&**d**, skin depigmentation, skin drying and atrophy, and scleroderma-like skin changes

The patient was a 26-year-old Chinese male with a chief complaint of impaired vision in both eyes for more than a year. An ocular examination revealed that the vision in his right eye was FC/20 cm and left eye was 0.02, intraocular pressure was 18 mmHg in both eyes, ptosis of both upper eyelids, lateral eyelashes touched the cornea. Corneas were transparent in both eyes, central corneal thickness was 547 μm in right eye, and left corneal thickness was 540 μm. The central anterior chamber depth of the right eye was 3.25 mm, and the central anterior chamber depth of the left eye was 3.03 mm. The pupils were round, about 3 mm in diameter. The lenses were milky and opaque in both eyes (Fig. 1a). The thickness of the right eye lens was 3.30 mm and the lens of left eye was 3.32 mm. The fundus of both eyes was not clear due to the occlusion of cloudy lens. The right eye axial length was 22.38 mm, and the left eye was 22.17 mm. No obvious vitreoretinal abnormalities were found on ultrosonography. Family history showed that his parents were consanguineous (first cousions). The patient’s father died in a traffic accident at 40 years old, and his mother, and sister, uncle, cousin, and niece were in good health. The patient denied any family history of genetic diseases. Developmental retardation occurred when he was 8 years old and Achilles tendon elongation was performed due to Achilles tendon contracture. Physical examination on admission revealed the patient had a spare figure, weighed 40 kg and was 150 cm tall (Fig. 2). Vital signs testing demonstrated his temperature was 36.7 °C, pulse was 98 b/min, respiratory rate 19/min, and the blood pressure was 108/65 mmHg. Heart and lung auscultation found no obvious abnormalities. A complete blood cell count, thyroid hormone levels, hepatic function, and renal function were evaluated, and no abnormalities were found. Figure 2 depicts the patient with the symptoms of short stature, slightly built, gray hair, bird-like face appearance, skin depigmentation, skin drying and atrophy, scleroderma-like skin changes, beak-like nose, and teeth abnormalities.

**Fig. 3:**
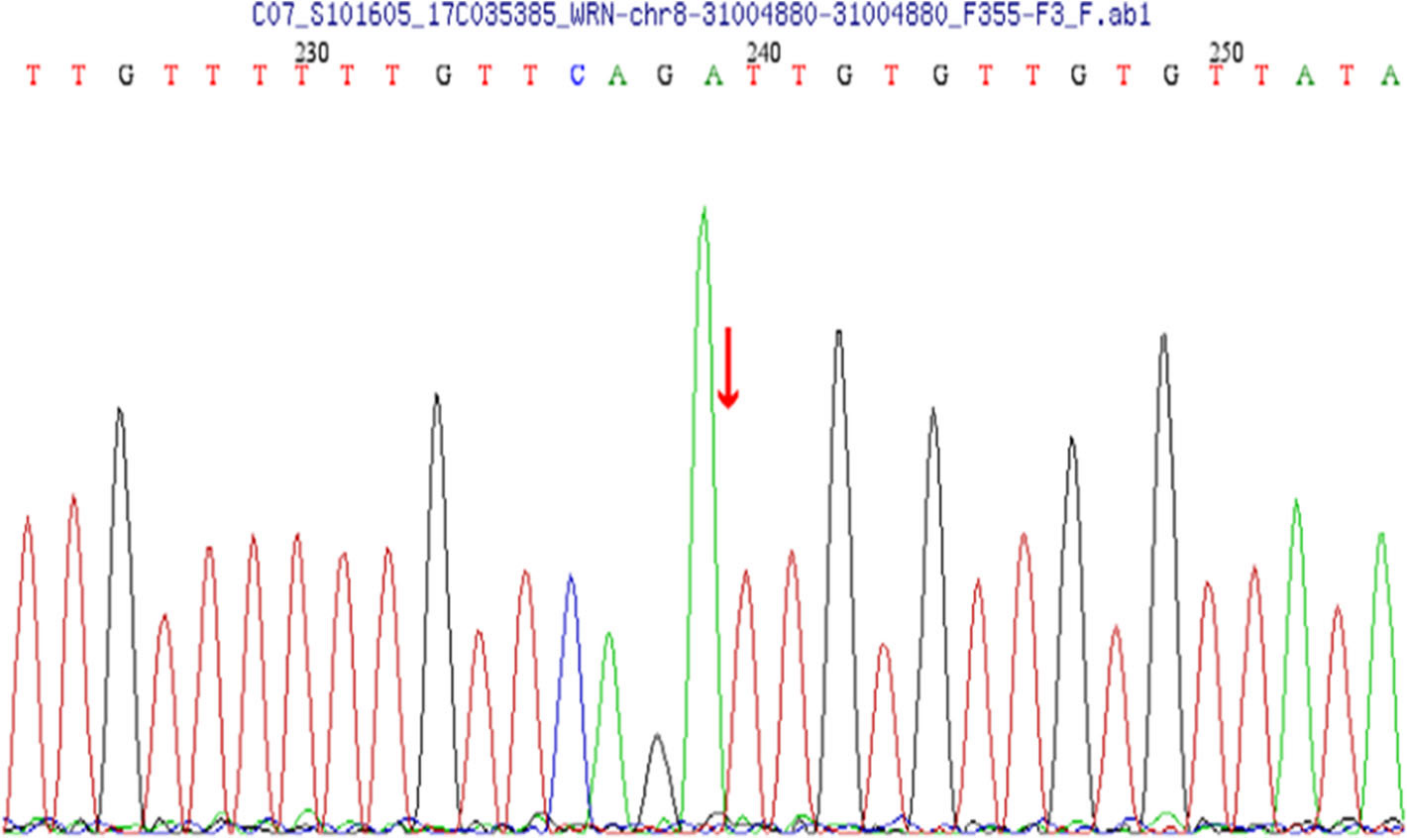
Next-generation sequencing identified a homozygous WRN mutation in this patient. Five bases (c.3460_3461 insTTGTG) were inserted between the 3460 and 3461 nucleotides of WRN gene in this patient

**Fig. 4:**
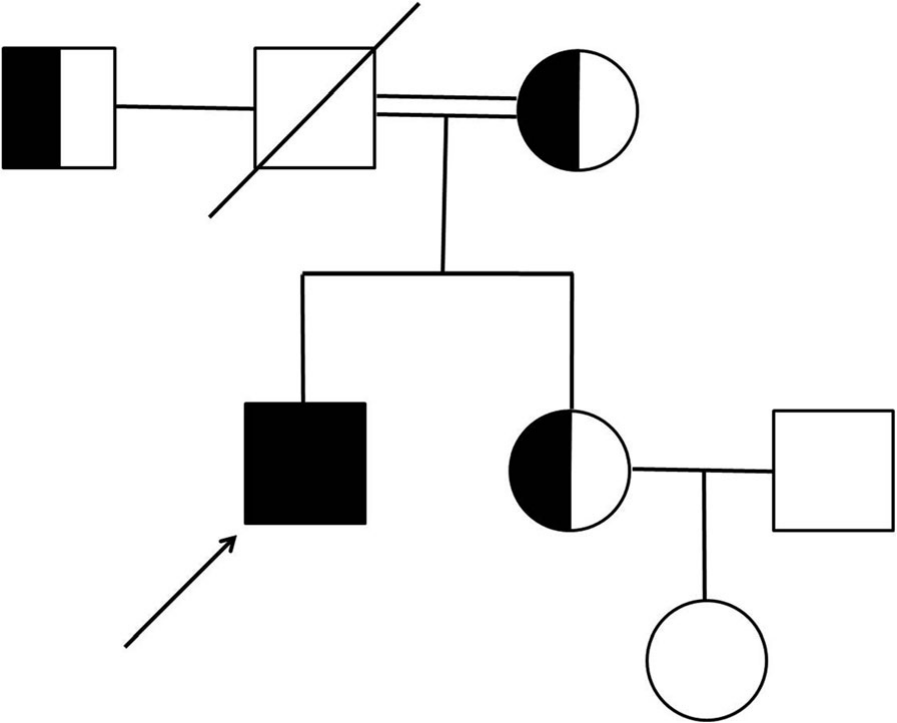
Pedigree chart of family showing disease across three generations of the disease

According to the ocular symptoms and systemic signs, including low body weight, a short stature, a bird-like face, atrophic and scleroderma-like skin, and juvenile cataracts, the clinical diagnosis of Werner’s syndrome was made. Next-generation sequencing identified a homozygous *WRN* mutation in this patient. Five bases (c.3460_3461 insTTGTG) were inserted between the 3460 and 3461 nucleotides of *WRN* gene in this patient, resulting in a frame shift mutation of amino acids (p. Y 1157 Cfs * 7) (Fig. 3). After searching the literature, this mutation has not been reported, and does not belong to the polymorphic site, incidence is very low in the population and not reported in the Human Gene Mutation Database (HGMD professional). The *WRN* mutation was the likely pathogenic variant. So the definitive Werner’s syndrome diagnosis was established. Besides, the same heterozygous *WRN* mutation was identified in his mother, his father’s brother, and sister. The pedigree chart has been constructed in Fig. 4.

The patient underwent phacoemulsification combined with intraocular lens implantation in both eyes. The right eye was implanted with a + 23.0 diopter intraocular lens and the left eye was implanted with a + 24.0 diopter intraocular lens. Post-operative appearance showed the clear IOL in place (Fig. 1b). The post-operative best-corrected visual acuity was 0.6 in both eyes. No vitreoretinal abnormalities were observed in both eyes.

## Discussion

The age-related pathophysiology resembles the normal aging [1]. Early susceptibility to a number of major age-related diseases is the key feature of this syndrome. The incidence of Werner’s syndrome is one in 20,000–40,000 in Japan and one in 200,000 in the USA [2]. However, the incidence of Werner’s syndrome has not been reported in China. Our case presented the characteristic of Werner’s syndrome resembling premature aging, including a bird-like face, atrophic skin, scleroderma-like skin, and juvenile cataract.

The definitive diagnosis for Werner’s syndrome is based on the genetic analysis for mutations in the *WRN* gene. *WRN* is a RecQ family member with both helicase and exonuclease activities and it participates in several cell metabolic pathways, including DNA repair, and telomere maintenance [3, 4]. The genetic pattern of this genetic disease is autosomal recessive. Homozygous mutation of *WRN* was detected, which may be inherited from their parents with consanguineous marriage. Homozygous *WRN* gene mutation (c.3460_3461 insTTGTG) was found in this patient and heterozygous *WRN* gene mutation were found in his close relatives. After physical examination, the relatives of the patient carrying the heterozygous mutations were not found to be associated with Werner’s syndrome. Features of early aging which include premature graying or loss of hair, juvenile cataract, type 2 diabetes mellitus, hypogonadism, osteoporosis, and atherosclerosis are very common in patients with Werner’s syndrome.

The patients with Werner’s syndrome develop a variety of serious diseases, especially atherosclerosis and malignant tumors. Early diagnosis of Werner syndrome is important to enable early serial screening for these associated diseases in the patients. Although there is no definitive therapy that addresses the underlying gene mutation, but many of the signs including cataracts, diabetes, and atherosclerosis are treatable. In our case, the patient was transferred to us due to his poor vision. After performing phacoemulsification and intraocular lens implantation in both eyes, his visual acuity improved significantly.

## Conclusions

Both the ocular symptoms and systemic signs combination with the homozygous WRN mutation indicated the definitive Werner’s syndrome diagnosis. Our case presented the characteristic of Werner’s syndrome resembling premature aging, including bird-like face, atrophic skin, scleroderma-like skin, and juvenile cataract. Genetic testing is important for the accurate diagnosis of inherited diseases and contributes to the early diagnosis and treatment of diseases.

## Abbreviation

HGMD: Human Gene Mutation Database
